# Necrotizing Gallstone Pancreatitis in a Pediatric Patient: A Case Report and Review of Diagnostic and Management Challenges

**DOI:** 10.7759/cureus.70802

**Published:** 2024-10-03

**Authors:** Amin Farsani, Allison Santi, Carlo Reyes, Pardeep Thandi, Arman A Sobhani

**Affiliations:** 1 Emergency Medicine, Los Robles Regional Medical Center, Thousand Oaks, USA

**Keywords:** acute necrotizing pancreatitis, gallstone pancreatitis, infected necrotizing pancreatitis, pediatric gastroenterology, severe pancreatitis

## Abstract

Necrotizing gallstone pancreatitis is a rare and severe form of pancreatitis, particularly uncommon in the pediatric population. While gallstone pancreatitis is increasingly recognized in children, necrotizing cases remain exceptional. We report a four-and-a-half-year-old Pakistani American male presenting with generalized weakness, abdominal pain, and vomiting. Initial symptoms followed a recent upper respiratory infection. Clinical evaluation revealed an intermittently drowsy-appearing patient with a Glasgow Coma Scale (GCS) of 15 when fully alert, hypotension, diffuse abdominal tenderness, and signs of possible sepsis. Laboratory tests showed elevated lipase levels and conjugated hyperbilirubinemia. Ultrasound identified gallstones and necrotizing changes in the pancreas, which were later confirmed by CT imaging. The patient was admitted to the pediatric intensive care unit for aggressive management of necrotizing pancreatitis, including fluid resuscitation, antibiotic therapy, and nutritional support. He underwent laparoscopic cholecystectomy and developed Clostridium difficile colitis, which was managed with targeted antibiotics. The patient had a 26-day hospital stay and was followed up with negative results from clinical exome sequencing for related disorders. This case underscores the diagnostic and management complexities associated with pediatric necrotizing gallstone pancreatitis. The need for a multidisciplinary approach and adherence to clinical guidelines is emphasized. This report contributes valuable insights into the rare presentation of necrotizing pancreatitis in children and highlights the importance of early and comprehensive intervention.

## Introduction

Gallstone pancreatitis, though relatively common in adults, presents a rare and challenging scenario in pediatric populations. Awareness and documented cases of pancreatitis among children are on the rise, with current estimates indicating a frequency of 1 in 10,000 for acute pancreatitis [1-3]. The occurrence of gallstone-induced necrotizing pancreatitis in children is particularly rare and noteworthy due to the infrequency of such cases in this age group. Necrotizing pancreatitis is estimated to affect less than 1% of children experiencing acute pancreatitis [4]. Among five extensive pediatric studies conducted in the United States, only 3 out of 1014 children exhibited necrotizing pancreatitis [5-9]. While there are a handful of case reports detailing necrotizing pancreatitis in children, information on its etiologies, progression, and results remains limited [10-14]. The scarcity of comprehensive studies in pediatrics necessitates an in-depth exploration of the clinical presentation, diagnostic challenges, and optimal management strategies in this unique context.

Gallstone pancreatitis arises from the obstruction of the common bile duct by gallstones, leading to inflammation of the pancreas. The intricate interplay between biliary obstruction, pancreatic inflammation, and the potential for secondary complications, such as peritonitis, requires careful consideration, especially in the pediatric population where the pathophysiological mechanisms may differ from those in adults.

Several studies highlight the atypical nature of gallstone pancreatitis in children, emphasizing the need for heightened clinical awareness and a thorough diagnostic approach. A study delves into the clinical spectrum of pediatric acute pancreatitis, outlining the challenges in differentiating it from other abdominal pathologies [15]. This study underscores the importance of a comprehensive diagnostic workup in pediatric patients presenting with acute abdominal pain.

## Case presentation

A four-and-a-half-year-old Pakistani American male with a past medical history of anterior fontanelle dermoid cyst status post excision and iron-deficiency anemia is brought in by his mother to the Emergency Department (ED) for generalized weakness, abdominal pain, and vomiting. The mother stated that one week prior to ED arrival, the patient was sick with symptoms of an upper respiratory infection, including fever and productive cough. The mother also stated that one day prior to ED arrival, the patient began developing abdominal pain with one episode of nonbilious and non-bloody emesis on the day of the ED visit. The patient’s mother noted that the patient had generalized weakness and had been sleeping more. The mother noted that the patient's fevers had subsided for the past three days but the patient still had intermittent productive cough. The mother stated that the patient has been eating normally with the last meal being last night. There is no reported recent travel or antibiotic use. The mother did report that the patient has had intermittent constipation over the past three to four days. The mother reported no shortness of breath, flank pain, dysuria, or decreased urination in the patient. The mother noted that the patient’s vaccinations are up-to-date.

On exam, the patient was afebrile (98.0 degrees Fahrenheit) with a pulse of 93 beats per minute, respiration of 20 breaths per minute, blood oxygen level of 93% on room air, and mildly hypotensive with a blood pressure of 89/58. The patient was intermittently drowsy but, when awake, was making eye contact and answering questions appropriately, with a Glasgow Coma Scale (GCS) of 15 when fully alert. No scleral icterus was observed on an exam. The pulmonary exam was normal. The patient had a soft, nondistended, diffusely tender abdomen without guarding, rigidity, or rebound tenderness. Murphy, Rovsing, and Obturator signs were negative, and there were no palpable masses or costovertebral angle tenderness. Differential diagnoses included acute gastroenteritis, gastritis, cholelithiasis, nephrolithiasis, and pancreatitis. There was still concern for sepsis secondary to appendicitis, cholecystitis, urinary tract infection, or pneumonia given the patient’s altered mental status, diffuse abdominal tenderness, and hypotension despite being afebrile. Thus, a sepsis and abdominal workup with orders for complete blood count, comprehensive metabolic panel, lipase, lactate, blood cultures, urinalysis, chest X-ray, and abdominal ultrasound were placed.

One-thousand four hundred (1400) milligrams of IV piperacillin-tazobactam to cover specifically for presumed intraabdominal infection, 500 milliliters of dextrose 5% in water/0.45% sodium chloride/10 milliequivalents potassium chloride IV solution and two doses of 1 mg of IV morphine were administered. Labs revealed a significant leukocytosis (30.3 10^3/microliters), thrombocytosis (466 10^3/microliters), elevated lipase (>30,000 units/liter (U/L)) and lactate (2.2 milligram/deciliter (mg/dL)), transaminitis (aspartate transferase: 230 U/L, alanine transferase: 248 U/L), acute renal insufficiency (creatinine: 0.46 mg/dL), and conjugated hyperbilirubinemia (total bilirubin: 2 mg/dL; direct bilirubin: 0.95 mg/dL). Labs revealed no anemia (hemoglobin 12.9 g/dL) but low mean corpuscular volume (70 femtoliters), normal calcium and triglyceride levels, and no growth on blood cultures (Table 1).

**Table 1:** Results of lab tests and their reference range AST: aspartate aminotransferase; ALT: alanine aminotransferase; MCV: mean corpuscular volume

Lab Test	Lab Value	Reference Range
Lipase	>30000 Units/L	48 - 199 Units/L
Lactic Acid	2.2 mmol/L	0.4 - 2.0 mmol/L
Triglycerides	57 mg/dL	Acceptable: < 75 mg/dL
Creatinine	0.46 mg/dL	0.17 - 0.42 mg/dL
Calcium	9.5 mg/dL	8.5 - 10.1 mg/dL
Calcium Adjusted for Albumin	9.7 mg/dL	8.5 - 10.1 mg/dL
Total Bilirubin	2.0 mg/dL	0.2 - 1.0 mg/dL
Direct Bilirubin	0.95 mg/dL	<0.3 mg/dL
AST	230 IU/L	10 - 47 IU/L
ALT	248 IU/L	16 - 61 IU/L
WBC	30.3 10^3/uL	5.0 - 13.0 10^3/uL
Hgb	12.9 g/dL	11.5 - 15.0 g/dL
MCV	70 fL	75 - 100 fL
Platelet Count	466 10^3/uL	150 - 400 10^3/uL
Blood Cultures	Negative	

**Figure 1:**
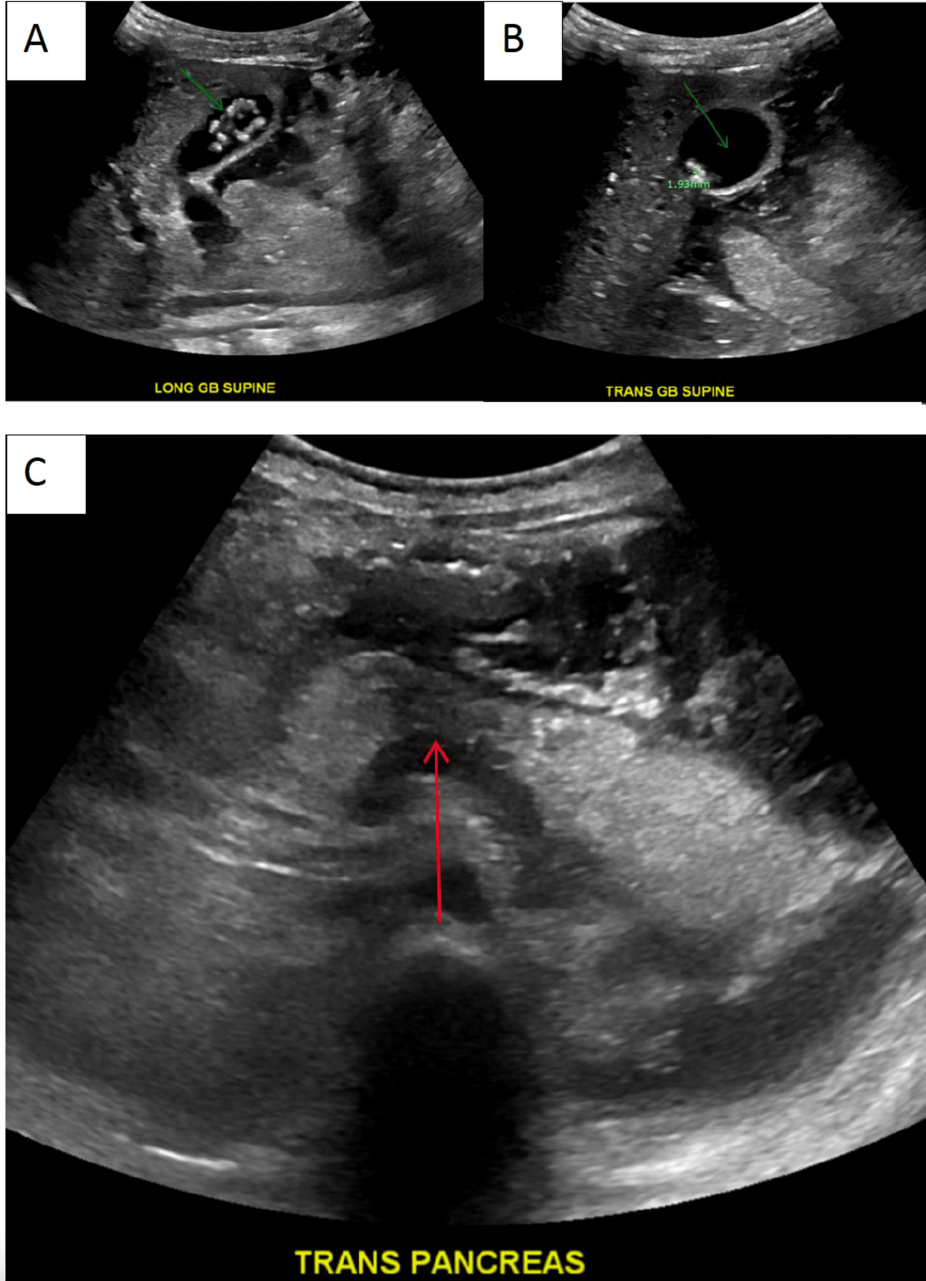
Ultrasound of the gallbladder and pancreas A) Long-axis view of the gallbladder revealing multiple gallstones (green arrow). B) Transverse view of the gallbladder (green arrow) revealing 2 mm stones (green measuring marker). C) Transverse view of the pancreas revealing cystic changes in the pancreatic body (red arrow) concerning for necrotizing pancreatitis.

Abdominal ultrasound revealed multiple 2 mm gallstones, normal common bile duct diameter, and cystic changes at the proximal body with a prominent tail (Figure 1). With the patient's significantly elevated lipase, conjugated hyperbilirubinemia, gallstones in gallbladder confirmed on ultrasound, and signs of necrotizing pancreatitis on ultrasound, the patient's diagnosis was consistent with necrotizing gallstone pancreatitis. On reassessment, the patient became more alert, and abdominal pain was controlled.

**Figure 2:**
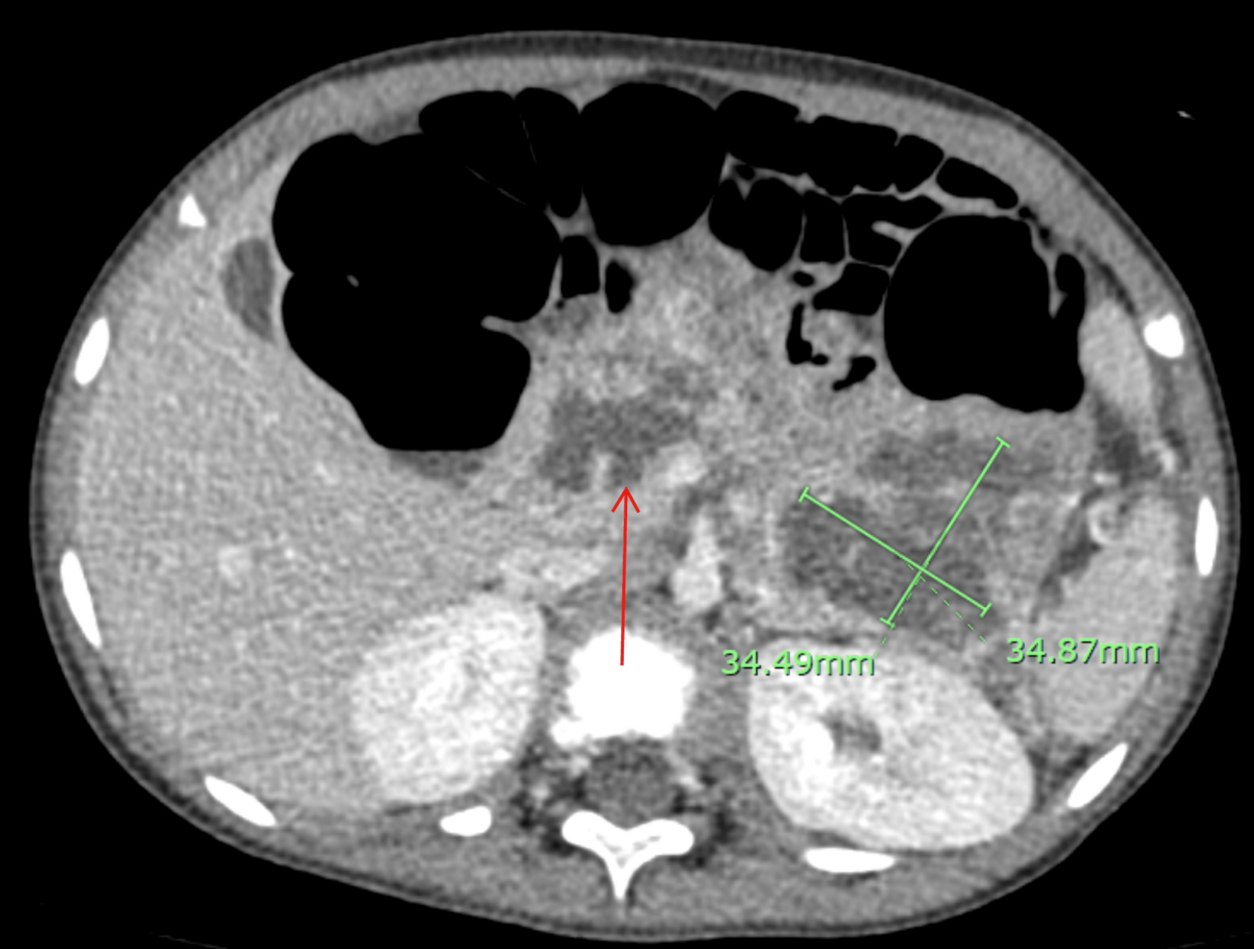
CT abdomen & pelvis with IV contrast revealing the remainder of the pancreas is almost entirely replaced by a loculated fluid collection without gas extending from the pancreatic head (red arrow) to the tail (green measuring marker), with either enhancement of parenchyma or peripancreatic tissue. This measures approximately 8 cm in length by 2 cm anteroposterior by 3.5 cm craniocaudal.

The patient was transferred to the pediatric intensive care unit for intensive medical management of necrotizing gallstone pancreatitis at one of the nearby pediatric hospitals and was hospitalized for 26 days. On hospital day (HD) two, enteral feeding via a nasogastric (NG) tube was initiated. The patient was started on a slow, continuous drip at 10 mL/hr of Peptamen Junior formula as tolerated. The enteral feeding was gradually advanced by 5-10 mL/hr every 12 to 24 hours as tolerated. On HD nine, the patient became febrile and CT abdomen/pelvis with IV contrast was ordered and revealed necrotizing pancreatitis with necrotic collections (Figure 2). On HD 11, due to persistent fevers, the patient was treated for presumed infected necrotizing pancreatitis with IV levofloxacin and metronidazole for one day and then transitioned to IV meropenem for the next eight days. On HD 17, the patient was able to transition from enteral feeding via an NG tube to a low-fat oral diet. On HD 19, he developed Clostridium difficile colitis and switched to IV metronidazole for five days. On HD 20, a laparoscopic cholecystectomy was performed. The patient followed up with a pediatric gastroenterologist and a clinical, focused exome sequencing analysis for iron-deficiency anemia and pancreatitis-related disorders was negative.

## Discussion

The diagnosis of pediatric acute pancreatitis (AP) should follow the International Study Group of Pediatric Pancreatitis: In Search for a Cure (INSPPIRE) criteria, requiring at least two of the following: compatible abdominal pain, serum amylase, and/or lipase levels three times the upper normal limit, or imaging findings consistent with acute pancreatitis [16]. The diagnostic modalities employed, including ultrasound, align with the recommended approaches outlined in the guidelines proposed by the Pediatric Pancreatitis Working Group [17]. Consensus guidelines recommend transabdominal ultrasound as the first imaging choice for children with suspected AP due to its availability, lack of ionizing radiation, and high sensitivity for gallstones and biliary obstruction, despite its moderate sensitivity for AP, which recent studies suggest is only 47-52% [18]. These guidelines provide a framework for the evaluation and management of pediatric pancreatitis, although specific considerations for gallstone pancreatitis in this age group remain limited. Therefore, this case report ultrasound played a role in identifying abnormalities consistent with necrotizing pancreatitis.

The management of gallstone pancreatitis includes early, aggressive fluid resuscitation, ideally with a lactated Ringer's bolus (10-20 milliliters/kilogram), careful monitoring, pain control, early enteral nutrition, and guidance on endoscopic or surgical interventions. Evidence for antibiotics and protease inhibitors is limited in this context [16]. The latest Surviving Sepsis Campaign guidelines discourage prolonged use of antimicrobial prophylaxis in patients with severe non-infectious inflammatory conditions, like severe pancreatitis, due to insufficient evidence supporting its effectiveness in preventing infections [19]. This rare condition in children often requires collaboration between pediatric gastroenterologists and surgeons to improve patient outcomes. A study highlights the importance of early intervention and a multidisciplinary approach, emphasizing timely treatment to prevent recurrent episodes and complications [3].

Gallstone pancreatitis has been linked to genetic factors. Several studies have identified specific genes that increase the risk of gallstone-related complications. A study identified gene variations related to lipid metabolism, gallbladder motility, and bile composition as key contributors to the heritability of gallstone disease [20]. A study provided a comprehensive analysis of the genetic predisposition to gallstones, highlighting the complex interplay between genetic factors and pancreatitis development [21]. This genetic understanding of gallstone pancreatitis provides valuable insights for developing targeted preventive measures and personalized treatment strategies, improving the precision and effectiveness of clinical management.

The triggers for progression from acute to necrotizing pancreatitis remain unclear. In adults, the severity of acute pancreatitis has been linked to genetic variations in antioxidant enzymes and glutathione depletion, which may contribute to the shift from mild to severe pancreatitis [22]. Another suggested mechanism involves reduced intravascular volume, inflammation, and elevated hematocrit, leading to obstruction of pancreatic blood flow and subsequent necrosis [4].

Necrosis worsens complications in acute pancreatitis. Therefore, a prompt CT scan with IV contrast is crucial after diagnosing acute gallstone pancreatitis to check for necrotizing pancreatitis, as done at the transferring facility. A 21-year retrospective study of children with necrotizing pancreatitis revealed that all were severely ill, requiring hospital stays between 9 and 40 days, with an average of 18 days [23].

Management strategies have shifted from immediate surgery to medical care, with surgery reserved for infected necrosis requiring specific treatment. Ongoing monitoring is crucial for detecting complications like pseudocysts and pancreatic insufficiencies [24].

## Conclusions

This case report underscores the rarity and complexity of necrotizing gallstone pancreatitis in children, highlighting the importance of including this severe condition in the differential diagnosis of acute abdominal pain and sepsis-like symptoms in pediatric patients. It emphasizes the need for a high index of suspicion and a multidisciplinary approach involving prompt imaging, targeted medical therapy, and potential surgical intervention. The case illustrates the value of comprehensive diagnostic workups and adherence to clinical guidelines for optimizing outcomes. Future research and case reports are crucial for improving understanding, management strategies, and clinical awareness of this rare condition in the pediatric population.
